# Research advances in the relationship between nonalcoholic fatty liver disease and atherosclerosis

**DOI:** 10.1186/s12944-015-0141-z

**Published:** 2015-12-03

**Authors:** Xin Xu, Linlin Lu, Quanyong Dong, Xiaolin Li, Nannan Zhang, Yongning Xin, Shiying Xuan

**Affiliations:** Department of Gastroenterology, Qingdao Municipal Hospital, Dalian Medical University, Qingdao, China; Digestive Disease Key Laboratory of Qingdao, Qingdao, China; Central Laboratories, Qingdao Municipal Hospital, Qingdao, China

**Keywords:** Nonalcoholic fatty liver disease, Atherosclerosis, Insulin resistance, Metabolic syndrome, Cardiovascular disease

## Abstract

Nonalcoholic fatty liver disease (NAFLD) is a metabolic stress-induced liver disease that is closely related not only to genetic susceptibility but also to insulin resistance and highly linked with metabolic syndrome. In recent years, the prevalence of NAFLD has increased rapidly, paralleling the epidemic of type 2 diabetes mellitus and obesity leading to cardiovascular disease. It has been demonstrated that NAFLD is highly associated with atherosclerosis. With recently gained knowledge, it appears that NAFLD may induce insulin resistance, dyslipidemia, oxidative stress, inflammation, and fluctuation of adipokines associated with atherosclerosis. In this review, we aimed to summarize recent discoveries related to both NAFLD and atherosclerosis, and to identify possible mechanisms linking them.

## Introduction

Nonalcoholic fatty liver disease (NAFLD) is a chronic liver disease that occurs in patients who consume little or no alcohol [1]. It has become one of the most common liver diseases worldwide and is characterized by parenchymal cell steatosis and steatohepatitis [2, 3], which can progress to cirrhosis, with or without liver failure and hepatocellular carcinoma (HCC) in a subset of patients [1, 4]. With increasingly sedentary lifestyle habits, NAFLD now has become the most common etiology of chronic liver disease in the US [5]. In Asian counties, the prevalence rates of 12 %–24 % [6] and in large cities in China with reported approximately 15 % [7]. NAFLD can occur at any age [8, 9]. NAFLD is closely associated with metabolic syndrome (MS), with nearly 90 % of NAFLD patients having more than one of the following conditions: obesity, type 2 diabetes mellitus (T2DM), hypertension, and dyslipidemia [10]. Because approximately 33 % of patients with NAFLD also have MS, NAFLD was widely considered to be the hepatic manifestation of MS [11, 12], but now more like a precursor of MS [13]. Although the pathogenic mechanism of NAFLD remains uncertain, the “two-hit” theory is widely accepted by us [14]. At present, Tilg, etc. have proposed the multiple parallel hits hypothesis. They suggested that inflammatory mediators derived from various tissues but especially from the gut and adipose tissue could play a central role in the cascade of inflammation, fibrosis, and finally tumor development [15].

Atherosclerosis (AS), a pathological plaque formation within blood vessels that initiates intimal thickening (the earliest lesion in the arterial wall [16]), hardening of the arteries, and narrowing of the lumen, is a leading causative factor for cardiovascular disease (CVD). Multiple phenotypes of AS disease based on pathological features and propensity for thrombosis have recently been proposed [17] according to the modified classification of American Heart Association (AHA) [18]. The classification includes intimal xanthoma, pathological intimal thickening, fibroadenoma, thin fibrous cap atheroma, and fibrocalcific plaque. The development of atheromatous plaques with a necrotic core represents an invasion of lipid deposits by macrophages. Release of activated proteolytic enzymes damages the surrounding tissues, leading to the formation of vulnerable plaque. The pathophysiology of AS is best described by “endothelial damage theory”, which implies that the components of AS, such as hypertension, dyslipidemia, etc., induce vascular intima injury to further stimulate the formation of atherosclerotic lesions. In addition, AS can become a systematic chronic inflammation involving many inflammatory cells and amboceptors [19].

### The association between NAFLD and AS

NAFLD is closely linked with MS [11–13], and is highly associated with abdominal obesity, atherogenic dyslipidemia, and diabetes. exposing subjects with NAFLD to an increased risk of CVD [20]. Studies have also shown that NAFLD is linked to CVD independent of other metabolic risk factors [21] as summarized in Table 1, suggesting that it is an active contributor to the pathogenesis of atherosclerosis and is not just a marker for CVD [22]. Various pathogenic mechanisms have been suggested as possible explanations for accelerated atherosclerosis and increased CVD burden in NAFLD patients, including a high oxidative stress state due to steatosis-stimulated fatty-acid oxidation in the liver [23], systemic release of proatherogenic molecules like tumor necrosis factor-α, interleukin-6, and oxidized LDL cholesterol [24], increased IR [25], and macrophage activation [26]. The atherogenic role of hepatic inflammation is also supported by the fact that patients with NASH have increased atherosclerosis when compared with patients with simple steatosis [27, 28]. Carotid intima-media thickness (CIMT) is a reliable index of subclinical atherosclerosis [29] and a mirror of atherosclerosis progression in NAFLD patients. Observational studies suggest that NAFLD is associated with increased CIMT and carotid plaques in both children and adults [30]. Therefore, NAFLD is closely associated with AS and it seems to an early risk factor for AS.

**Table 1:** Clinical studies about the link between NAFLD and AS

Author, year	NAFLD/NASH is associated with AS-related phenotypes
Mishra S, 2013 [115]	NAFLD is associated with carotid intima-media thickness
Li N, 2014 [116]	NAFLD is associated with carotid artery wall thickness
Colak Y, 2012 [117]	
Sunbul M, 2014 [118]	
Kozakova M, 2012 [119]	NAFLD as estimated by the fatty liver index is associated with early carotid plaques in middle-aged nondiabetic subjects
Yilmaz Y, 2010 [120]	NAFLD is associated with decreased coronary flow reserve
Pacifico L, 2010 [121]	NAFLD is associated with reduced brachial artery flow-mediated vasodilation and increased carotid artery wall thickness
Alkhouri N, 2011 [122]	NAFLD is associated with increased arterial stiffness and reduced brachial artery flow-mediated vasodilation
Villanova N,2005 [123]	NAFLD histology is associated with reduced brachial artery flow-mediated vasodilation
Colak Y, 2012 [117]	
Ampuero J, 2015 [124]	NAFLD is associated with carotid intima-media thickness and carotid plaques (meta-analysis of 14 studies involving 4130 subjects)
Guleria A, 2013 [125]	NAFLD is associated with carotid intima-media thickness and flow-mediated dilatation %
Torun E, 2013 [126]	
Chen Y, 2015 [127]	NAFLD is associated with carotid intima-media thickness and brachial-ankle pulse wave velocity in patients with advanced fibrosis
Ozturk K, 2015 [128]	NAFLD is associated with PWV, CIMT and FMD levels in young adult men
Pastori D, 2015 [129]	NAFLD is associated with FMD level in patients with cardiometabolic risk factors.
Puiq J, 2015 [130]	NAFLD is associated with CIMT in morbid obesity
Kim SK, 2014 [131]	Nonalcoholic Fatty liver disease is associated with increased carotid intima-media thickness only in type 2 diabetic subjects with insulin resistance
Kucukazman M, 2013 [11]	NAFLD is associated with CIMT and FMD levels

Generally, the increased risk of CVD in NAFLD patients [31, 32] might reflect the coexistence of MS components. This may suggest that NAFLD confers a cardiovascular risk above or even beyond its association with the individual components of MS [33]. The high prevalence of NAFLD in AS patients has stimulated an interest in the possible role of the liver in the development of AS. Therefore, identification of the mechanism linking NAFLD and AS may be helpful in the development of a therapeutic target in AS [34] and in the prevention and treatment of CVD in early NAFLD patients.

### Possible mechanisms linking NAFLD and AS

NAFLD can contribute to and aggravate AS development, but the precise mechanism remains unclear. The following display possible linkages between these conditions at the molecular level.

#### Insulin resistance (IR)

IR, as the “first-hit” to the liver, contributes to the development of both NAFLD and AS by disrupting cellular energy metabolism, damaging the peripheral tissue, interfering with the ingestion and synthesis of liver fatty acid, and promoting fatty acid accumulation in the benign liver, which leads to hepatic IR due to a lack of suppression of endogenous liver glucose production [35]. NAFLD patients with IR experienced additional stresses to the liver based on the presence of hyperglycemia, hyperinsulinemia, hyperlipidemia, and damage to the vascular endothelial cells (VECs). All of these factors participate in the development of AS. Furthermore, increased VEC adherence can induce proliferation of smooth muscle cells (SMCs) and promote the synthesis and release of growth and inflammation factors in various pathways, which contributes to the progression of AS.

#### Dyslipidemia

Given that the regulation of lipid influx, synthesis, and metabolism is disturbed in the liver of NAFLD patients, NAFLD is associated with dyslipidemia, which leads to an up-regulation of the sterol regulatory element binding protein-1c (SREBP-1c), a transcription factor for some de-novo lipogenesis genes, to inhibition of the free fatty acid (FFA) oxidation and stimulation of liver fat content (LFC) [35–37]. Likewise, SREBP-2 and low-density lipoprotein (LDL) receptor are down-regulated in NAFLD patients, leading to inhibition of cholesterol uptake and very low-density lipoprotein (VLDL) synthesis in liver cells, resulting in an increase in hepatic triglycerides (TG) [36]. Increased TG levels can further disturb the atherogenic lipid profile by lowing high-density lipoprotein cholesterol (HDL-C) (an anti-AS factor) [37] and increasing small and dense LDL particles and oxidated LDL (ox-LDL) [38]. Ox-LDL contributes directly to AS and accelerates the development of local atherosclerotic plaques [39] as a key molecular connection between NAFLD and AS. Furthermore, as blood FFAs are increased due to increased energy intake and decreased FFA oxidation [40], endothelial cells (ECs) can be affected morphologically with shrinkage and intercellular space dilatation. With an increase in cellular permeability, serum remnant-like particle cholesterol (RLP-C) can easily gain access into ECs and block and interfere cellular activity, leading to AS [41]. Additionally, FFAs could disturb the insulin level by inhibition of its gene transcription through Jun N-terminal kinase (JNK) [42] to contribute to IR and promote the development of AS.

#### Oxidative stress and lipid peroxidation (LPO)

Oxidative stress and lipid peroxidation (LPO), as the “second-hit” to the liver in the development of NAFLD, may be another important mechanism linking NAFLD with AS. Oxidative stress is an imbalanced situation in which the body’s production of reactive oxygen species (ROS) exceeds its capability for ROS detoxification, causing tissue damage [43]. ROS can be produced by increased activity of reduced nicotinamide adenine nucleoside phosphate (NADPH) oxidase through activation of phosphate kinase C (PKC) or by increase of β-oxidation of peroxisomes and ω-oxidation of microsomes. It induces levels of inflammatory factors [44], depletes NO [45], destroys the endothelium-dependent vasodilatation function [46], reduces the elasticity of blood vessels, promotes endothelial cell apoptosis [47], contributes to vessel smooth muscle cell hyperplasia [48, 49], causes endoplasmic reticulum (ER) stress, leads to hyperlipidemia, promotes apoptosis of macrophages in the atherosclerotic plaque, and induces ox-LDL through LPO [50, 51]. All of these metabolic derangement clearly indicate that NAFLD is potential strongly associated with AS.

#### Inflammation

Inflammation can induce IR [52], whereas reduction of inflammation prevents IR development [53]. In NAFLD patients, inflammation appears as an increases in the levels of cytokines interleukin (IL)-6 and tumor necrosis factor (TNF)-α, C-reactive protein (CRP), and monocyte chemoattractant protein-1 (MCP-1) in peripheral blood [54]. Increased hepatic expression of IL-6 and increased blood levels of IL-6 may promote partial liver injury and AS [51]. IL-6 can activate macrophages to secrete matrix metalloproteinase-1 (MMP-1), induce mononuclear cells (MNCs) to participate in the development of vessel plaque, promote synthesis of LDL receptor and influx of LDL into macrophages, enhance lipid deposition, and stimulate vascular smooth muscle cell (VSMC) proliferation [55]. Inflammation and IR, therefore, participate in the development of AS [56].

#### Matrix metalloproteinase (MMP)

MMPs, a main enzyme family involved in degradation of extracellular matrix, is secreted predominantly by MNCs, macrophages, and VSMCs. An increase in MMP expression is detected in NAFLD patients [57], and this increase may play a role in the course of liver fibrosis and in the process or fracture of AS by degradation of fibrous cap disruption of plaque and promotion of thrombus formation preceded by formation of a vulnerable atherosclerotic plaque.

#### Levels of Adipokines

Adiponectin (APN), which is secreted by adipocytes, can enhance insulin sensitivity in the liver and other tissues to reduce the level of serum fatty acids and increase the oxidation of fatty acids in the muscle [58]. It has been found that APN levels are low in NAFLD patients independent of metabolic disorder [54]. Althouth an increase in APN expression can reduce TG, total cholesterol, and LDL-C concentrations [59], stimulate vascular endothelial nitric oxide synthase (eNOS) mRNA expression, and progressively reduce atherosclerotic lesions. The possible mechanism may involve increases in superoxide dismutase (SOD) and eNOS activities as well as APN expression, a decrease in MDA levels [60], and inhibition of macrophage scavenger receptor A1 expression and transformation to foam cells, inhibition of VSMC proliferation and migration to suppress plaque disruption, and an increase in competitive binding between platelet growth factor (PDGF) and the BB receptor to suppress signal transduction. It has been demonstrated that APN inhibits the EC inflammatory reaction by affecting the nuclear factor (NF)-κB signaling pathway [60, 61], another key molecular pathway involved in AS.

Leptin, which is predominately produced by adipose tissue, plays an important role in regulating food intake and energy expenditure and has been found to be elevated in NAFLD patients [60] in association with disease severity [62]. In addition, leptin plays a crucial role in aggravation of NASH [63, 64] and attenuation of AS. Endogenous leptin resistance is associated with IR with a synergistic effect. It can surpress apolipoprotein M and APN levels, reduce NO synthesis by activating the PI3-Akt-eNOS pathway [65], stimulate IMT, and serve as a predictor of CVD [66].

Visfatin, a new adipocytokine expressed in visceral adipose tissue (VAT) and with nicotinamide phosphoribosyltransferase (NAMPT) activity, is associated with some of AS risk factors such as inflammation, endothelial dysfunction, vascular endothelial proliferation, and atherosclerotic plaque formation. Visfatin has been suggested as a new indicator of the severity of NAFLD, as its expression is closely and positively associated with the degree of liver steatosis [67] and increased in the foamy macrophages of AS plaques. Overexpression of visfatin induces foamy macrophages to secrete MMP [68]. Therefore, visfatin may be involved in the processes of both AS and NAFLD.

Chemerin, a chemoattractant protein, is highly expressed in the white adipose tissue (WAT) and liver of NAFLD patients and functions to attract macrophages and immature dendritic cells (DCs) via binding with a chemotaxis receptor. It has been suggested that chemerin may be closely related to risk factors of CVD (such as hypertension) [69] and play a critical role in the development of both NAFLD and AS, although the precise mechanism still needs to be investigated.

Omentin, a cytokine expressed in omental adipose tissues, has an anti-inflammatory effect and can increase insulin sensitivity. It also has a vascular relaxation effect via the regulation of NOS expression [70]. A significant reduction in omentin expression in NAFLD patients may imply a connection between omentin and AS.

Retinol-binding protein 4 (RBP4), a specific retinal transfer protein, is compounded and secreted by liver cells for binding and transportation of retinal from blood into cells. RBP4 expression is increased upon liver injury, NASH (as a sensitive maker of NASH) [71], or coronary heart disease (especially with acute coronary syndrome [ACS]) [72] and thus, has been proposed to be a new risk factor for coronary heart disease [73]. Accumulating evidence suggests that RBP4 can inhibit insulin activity in ECs, weaken the level of NO, and promote endothelial dysfunction [74] in the development of AS. Furthermore, gene expression of RBP4 has become a sign of inflammation [25–76] in association with AS. However, the detailed molecular interaction that occurs in the progression of NAFLD and AS remains to be elucidated.

Resistin is closely associated with NAFLD and AS [77] via the following possible mechanisms: (1) resistin causes vascular endothelial dysfunction by increasing the release and expression of vascular cell adhesion molecule-1 (VCAM-1), intercellular adhesion molecule (ICAM)-1, and pentraxin-3 (PTX-3); by decreasing tumor necrosis factor receptor-associated factor (TR-AF)-3 expression to induce CD40 and MCP-1 expression; by inducing P selectin expression to stimulate NADPH oxidase activity; and by enhancing the adhesion of monocytes as well as NF-κB and activator protein-1 activities [78]. (2) NF-κB and peroxisome proliferator activated receptor-y (PPARy) can regulate resistin secretion, and the NF-κB signaling pathway plays an important role in the progression of AS with the participation of resistin [79]. (3) Resistin promotes SMC proliferation by activating the corresponding signaling transduction pathway [80, 81], enhances the migratory ability of SMCs [82], and induces oxidative stress and an inflammatory reaction under dyslipidemia. Therefore, resistin is involved in NAFLD and acts as an indicator of the severity of AS [83].

#### Intestinal microbiota

A great deal of data have shown that the intestinal microbiota was a risk factor of contributing to the development of NAFLD [84]. The mechanism underlying may as follows: the level of lipopolysaccharide (LPS) derived by intestinal microbiota was increased in NAFLD patients, and when increased plasma endotoxin concentration in the portal vein, the clearance ability of the hepatic Kupffer cells may become overloaded [85], leading to systemic endotoxaemia and mild chronic inflammation [86], inducing chronic liver disease [87]. Indeed, numerous studies support a complicated relationship between the intestinal microbiota and obesity. Obese individuals are thought to have increased the intestinal permeability [88], potentially inducing an increased endotoxin load in the portal vein and ensuing overload of the hepatic Kupffer cells. Obesity and NAFLD are important risk factors for the development of AS and subsequent CVD. In fact, intestinal microorganisms also seem to be involved in AS. Study showing that plasma endotoxin levels above 90 percent were linked with a three-time increase in cardiovascular event risk [89], and animal experiments clearly indicating that endotoxin injection accelerates cholesterol-induced AS [90]. Besides, numerous studies have showing intestinal microbiota was closely link with IR, which is the common mechanism of NAFLD and AS.

#### Fetuin-A

Fetuin-A, a a-2-HS-Glycoprotein, is a multifunction-protein synthesized in the liver and secreted into the circulation [91]. It is not only an endogenous inhibitor of insulin receptor tyrosine kinase in the skeletal muscle, but in the liver [92] resulting in IR. Furthermore, Pal et al. currently showed that Fetuin-A acts as an endogenous ligand for toll-like receptor 4 (TLR4) and enhances both IR and inflammation [93]. High level of Fetuin-A is closely associated with IR, atherogenic dyslipidemia, elevated inflammatory cytokines, and decreased adiponectin levels [94]. These findings suggest that Fetuin-A may contribute to both the course of NAFLD and development of AS, and it is increased in NAFLD subjects. However, this glycoprotein inhibits ectopic calcification, a reduction level of Fetuin-A might promote cardiovascular calcifications [94]. Moreover, this glycoprotein is an inhibitor of transforming growth factor-b1 (TGF-b1), a major pro-fibrogenic growth factor promoteing fibrotic changes in the liver and arteries [95]. As mentioned above, we may reasonable speculate that higher level of fetuin-A could prevent NAFLD and AS development. Despite the function of Fetuin-A for NAFLD and AS remains controversial, but it may be a link between NAFLD and AS.

#### Obstructive sleep apnea (OSA)

OSA is one of the most common types of sleep apnea [96] and is closely related to NAFLD (sharing it with similar risk factors) [97] and early-stage AS, as determined by the CIMT and pulse-wave velocity (PWV) [98, 99]. Increased CIMT was found in OSA patients [100], and OSA is pathologically related to CVD and AS [101]. The possible mechanistic links between AS and OSA may be intermittent hypoxia and oxidative stress, the inflammatory cascade, endothelial dysfunction, mechanical and hemodynamic factors, and platelet activation and coagulation abnormalities [102].

#### Heart-type fatty acid binding protein (H-FABP)

H-FABP, a cytosolic protein, transports fatty acids in cardiomyocytes. It regulates the mitochondrial beta-oxidative system within cardiomyocytes and accounts for 10 % of cytosolic protein in these cells [103]. Serum H-FABP is highly sensitive to myocardial ischemia and used as a diagnostic biochemical indicator of ACS [104]. H-FABP is closely related to NAFLD and AS. H-FABP levels are elevated in NAFLD patients and significantly and positively linked to the CIMT (the early marker of subclinical AS) [105].

#### Chronic kidney disease (CKD)

It has been discovered that NAFLD is positively associated with CKD [106–108]; however, the possible mechanism remains vague. Both NAFLD and CKD can increase the risk of CVD [109, 110]. Obviously, a CKD-specific bone mineral disturbance can strongly induce the calcification of plaques and substantially promote AS development. Therefore, NAFLD may affect or accelerate the development of CVD with CKD [111].

#### Others

Markers of fibrinolytic and hemostatic function (such as plasminogen activator inhibitor-1 antigen) are closely linked with NAFLD, AS, and CVD [112, 113].

## Conclusion

NAFLD is a metabolic stress-induced liver disease that is closely related to IR and highly linked with MS. In recent years, the prevalence of NAFLD has increased rapidly with a higher concurrence in the patients with T2DM and AS leading to CVD. NAFLD has emerged as a public health problem worldwide and is highly associated AS, It has been demonstrated that NAFLD leads to an increased risk of cardiovascular events and mortality [114]. The mechanism linking NAFLD and AS is poorly understood and may be related to IR, inflammation, oxidative stress, lipid disorders, MMP activity, fatty hormone levels, CKD, and OSA. We discussed and summarized the available evidence in an attempt to reveal the mechanistic connection between these two common pathological conditions.
